# Mr. Nunn's Case of Poison

**Published:** 1803-03-01

**Authors:** R. Nunn

**Affiliations:** Pupil to Mr. Newell. Colchester


### *To the Editors of the Medical and Physical Journal.*

GENTLEMEN,

CONCEIVING that accounts of cases similar to the one I am going to lay before you, are not very frequent, and that the modes of preventing, or rather obviating the effects of active poisons introduced into the stomach, are not very generally understood, either from their infrequency, or practitioners of medicine not being called in sufficient time to venture upon the exhibition of remedies: I have taken the liberty of submitting one I was called to the other day, to your perusal; and wish, if you think it sufficiently important, it may have a place in your very useful and widely circulated Journal.

On the 31st of January last, I was called to Mary Ryford, aged twenty-four, of this town, whom the messenger

 represented represented to be dying, and in violent agonies. On visiting her, I found she was perfectly senseless, her pulse slow and feeble, extremities cold, her eyes staring, with the pupils much dilated, and discharging tears profusely, her countenance extremely swelled and bloated.

Upon enquiry, I found that she had about two hours previous to my seeing her, swallowed ten or twelve pills, which had been given her by a young man for the cure of syphilis. I immediately made application to the druggist, who had prepared them; and desiring to see the prescription, I found that each pill contained half a grain of the *oxygenated muriate of mercury*, and the same quantity of gamboge. Having obtained this information, I proceeded, agreeably to the account of some cases I had recollected to have seen, to give her some kali, of which I dissolved half an ounce in two ounces of water, and gave her a spoonful as frequently as I conveniently could; but I was obliged to keep the handle of a knife between her teeth, as otherwise they were so closely locked as to preclude my doing any thing. I continued this treatment for the space of an hour and a half, when a slight vomiting with a diarrhœa came on, and I was happy to perceive that she continued from that time to recover, although she complained much of severe pains in her stomach, terrible heat in her throat, constant cough, &c. But these symptoms gave way to warm, soft gruel, solutions of gum arabic, and diluting drinks of a similar nature, so that in a few days she was perfectly recovered.

This case amongst too many others, shews the horrid ills resulting from a practice which is daily increasing in this country, viz. that class of men under the denomination of druggists being permitted to make up, vend, and *even prescribe* medicines for the cure of diseases. It is true this young woman took the pills in a moment of despair; but we cannot say that they were prepared correctly, and we are certain that the dose was much beyond what is common to prescribe of so powerful and violent a medicine; she was directed to take one, night and morning.

I am, &c.

R. NUNN,
Pupil to Mr. Newell.

*Colchester, Feb. 8, 1803.*

